# Please Don’t Look at Me That Way. An Empirical Study Into the Effects of Age-Based (Meta-)Stereotyping on Employability Enhancement Among Older Supermarket Workers

**DOI:** 10.3389/fpsyg.2019.00249

**Published:** 2019-02-22

**Authors:** Pascale Peters, Beatrice I. J. M. Van der Heijden, Daniel Spurk, Ans De Vos, Renate Klaassen

**Affiliations:** ^1^Institute for Management Research, Radboud University Nijmegen, Nijmegen, Netherlands; ^2^Expertise Center Strategy, Organization and Leadership, Nyenrode Business Universiteit, Breukelen, Netherlands; ^3^Faculty of Management, Science and Technology, Open University of the Netherlands, Heerlen, Netherlands; ^4^Kingston Business School, Kingston University London, London, United Kingdom; ^5^Business School, Hubei University, Wuhan, China; ^6^Institut für Psychologie, University of Bern, Bern, Switzerland; ^7^Antwerp Management School, University of Antwerp, Antwerp, Belgium

**Keywords:** career development, diversity climate, employability, HRM, (meta-)stereotyping, older workers

## Abstract

At present, individuals increasingly have to take ownership of their working lives. This situation requires them to self-manage and plan their careers. However, individuals’ career management does not happen in a vacuum. Studies have therefore stressed the importance of organizations introducing Sustainable Human Resource Management to share the responsibility for individuals’ employability. This is expected to motivate especially disadvantaged workers, such as older workers (≥ 50 years) and those working in lower-skilled jobs, to work longer across the life-span. In view of the growing scholarly and societal attention for Sustainable Career Development (SCD), the present study examines the relationships between workers’ chronological age (comparing older workers with younger and middle-aged groups, respectively) and dimensions of self-reported employability, and how perceptions of negative (meta-)stereotyping regarding older workers’ productivity, reliability, and personal adaptability moderate these relationships. To examine how possible underlying psychological mechanisms can affect individuals’ labor market decisions and behaviors, we developed hypotheses derived from socio-emotional selectivity and self-categorization theory, which we tested using data collected among supermarket workers in various age groups (*N* = 98). Moderated regression analyses showed that, in line with our hypotheses, perceptions of negative age-based (meta-)stereotyping amplifies the negative effect of older workers’ age on their self-perceived employability. In particular, we found that: (1) the older worker group reported lower levels of three of the distinguished employability dimensions (i.e., anticipation and optimization, corporate sense, and balance, but not occupational expertise and personal flexibility) and (2) perceptions of stronger negative (meta-)stereotypes regarding older workers in the organization had a moderating effect on the relationship between age group and four of the distinguished employability dimensions (i.e., occupational expertise, anticipation and optimization, corporate sense, and balance, but not personal flexibility). We conclude that age group membership as well as negative age-based (meta-)stereotypes deter older workers from enhancing their employability, which may potentially impact their career decisions and opportunities, especially in view of swift changing labor market demands. We argue, therefore, that Sustainable HR practices should focus on opposing negative age-based (meta-)stereotyping and on creating an inclusive work climate, meanwhile enhancing workers’ ambitions and career opportunities over the life cycle.

## Introduction

De Prins et al. (2015) notion of Sustainable Career Development (SCD) emphasizes the need for *respect* for internal organizational stakeholders (i.e., workers); *openness* or environmental awareness, including an outside-in and inside-out perspective on Human Resource Management (HRM); and *continuity* or a long-term approach to economic and societal sustainability and to employability enhancement, an important condition for individuals’ financial self-sufficiency (De Vos et al., 2016). In this study, we particularly focus on employability, viewed as an important factor in SCD, as this capacity enhances individuals’ labor market opportunities (Van der Heijde and Van der Heijden, 2006). Employability has become an important topic, especially in view of a combination of several trends, such as the increasing ageing and dejuvenization of the workforce (Shultz and Adams, 2012), the associated rise of the legal age of retirement and, hence, the need for individuals to work longer over the life course. In this context, it is also worth mentioning the fierce competition in globalizing markets, driven by technological change, such as ongoing automation, which demands both organizations and individuals to become more agile and adaptable (Brynjolfsson and McAfee, 2011). In fact, those workers who don’t adapt well may experience social exclusion (De Vos and Van der Heijden, 2015), which may also have severe financial consequences for them. In order to motivate and enable individuals to take ownership of having a sustainable career and to be financially self-sufficient, organizations may need to develop HR strategies that both stimulate and support longer working over the life cycle.

SCD focusses on workplace inclusion of all workers (De Prins et al., 2015), which may require organizations to pay particular attention to disadvantaged worker categories who are otherwise at risk of being excluded from organizational career support. It has been acknowledged, for example, that the employability of older workers in various work environments, and how this is being influenced by psychological mechanisms, demands more attention (cf. Froehlich et al., 2015). Particularly in view of the trends mentioned above, maintaining and enhancing older workers’ employability to motivate and enable them to work longer over the life cycle (cf. Dordoni et al., 2017) is a challenge for all types of working organizations. However, this may especially hold true for organizations employing older workers in lower skilled work (Frey and Osborne, 2013), such as those being in jobs characterized by manual and routine-based service work. Their tasks and responsibilities may be subject to technological developments, which make their work redundant, demand for different skill sets, and ask for higher levels of adaptability. Therefore, this study addresses older workers holding lower-skilled jobs.

More specifically, this study examines the impact of workers’ age group membership [(younger (< 30 years old); middle-aged (30 to 49 years old); and older (50 to 67 years old)] on their self-reported employability, and how the alleged negative relationship between older age group membership and self-reported employability may be amplified by perceptions of negative (meta-)stereotyping regarding older workers (cf. Henkens, 2005). We build on two theories: socio-emotional selectivity theory (SST) (Carstensen et al., 1999; Carstensen, 2006) and self-categorization theory (Turner, 1982; Tajfel and Turner, 1986). First, Socio-emotional Selectivity Theory (SST) comprises a useful framework since it focuses on changes in workers’ perceptions of time and future opportunities (in their work and in other life spheres) which may result in changes in their motives to engage in social interaction. More specifically, SST expects that over the life cycle workers will become more intrinsically motivated (e.g., being more focused on affective goals, such as generativity, emotional intimacy, and feelings of social embeddedness) and less extrinsically motivated (e.g., being less focused on instrumental goals, such as social status, social acceptance and professional learning and development) (Lang and Carstensen, 2002; Akkermans et al., 2016). The more “limited time perspective” that is experienced by older workers can therefore be expected to have consequences for their motivation to invest in future employability and career-enhancing activities. Second, self-categorization theory (SCT) is also a useful theory in the light of our study as it addresses the processes by which people form cognitive representations of themselves and others in relation to different social groups (Turner, 1982; Tajfel and Turner, 1986) and helps to understand why (meta-)stereotyping may occur. Stereotyping is a well-known phenomenon which implies that outgroup members (e.g., younger workers) use systematic cognitive generalizations about individuals which are based on the ingroup to which those individuals belong (e.g., older workers) without taking into account possible differences across individuals within that group. Although this may be an efficient way to make judgments about the ingroup members’ attitudes and behaviors, it can lead to biased judgments (Nahavandi et al., 2015). In a related vein, ingroup members’ beliefs with regard to the outgroup members’ cognitive generalizations regarding the ingroup (e.g., older workers) can be referred to as “meta-stereotypes” (Vorauer et al., 1998; Owuamalam and Zagefka, 2011, 2014).

Based on the outcomes of our empirical analysis, we conclude by presenting recommendations for SCD that enable key parties (i.e., direct supervisors, top management, and HRM specialists) in organizations to oppose negative effects of (meta-)stereotyping in general, and age-based (meta-)stereotyping in particular. We will also address implications for the important role that might be played by individuals themselves and their line managers, and will go into the role of social dialogue, in particular in the light of SCD across the life-span.

## Theory and Hypotheses

### Conceptualizing Employability and Its Dimensions

The definition for employability adopted in this study comprises individuals’ “capacity of continuously fulfilling, acquiring, or creating work through the optimal use of competences” (Van der Heijde and Van der Heijden, 2006, p. 453). This capacity enables individuals to create, maintain, or find employment within or outside their current work contexts (Van der Heijde and Van der Heijden, 2006). In this study, we focus on individuals’ self-perceived employability, as individuals’ perceptions are the main drivers of their behavior (Katz and Kahn, 1978). To remain employable and ensure life-long employment and personal career success (De Vos et al., 2011), individuals need to continuously focus on and develop those competences that are needed in the labor market, which may go beyond their domain-specific expertise. Therefore, individuals also need to develop more general competences which allow them to be proactive and flexible, cope with ambiguity, and manage multiple tasks. These competences correspond to what Hall (2004), in his Protean Career theory, refers to as “meta-competences” which are important for continuous learning and enable individuals to stay employable. Since employability can therefore not be seen as a unidimensional construct, in line with Van der Heijde and Van der Heijden (2006), in this study five competence-based employability dimensions are distinguished.

The first dimension relates to domain-specific *occupational expertise* (i.e., knowledge, skills, including meta-cognitive ones, and social recognition by relevant others), whereas the other four dimensions, *anticipation and optimization*, *personal flexibility*, *corporate sense*, and *balance*, relate to more general, job and career related competences. Anticipation and optimization as well as personal flexibility refer to individuals’ competence to adapt to changing labor market needs. The first dimension, specifically, refers to the individual being proactive and creative in adjusting to changing (internal or external) labor market needs, whereas the second dimension refers to the individual being more passive and reactive in this regard. Corporate sense refers to individuals’ social competences, such as displaying team and organizational commitment and network activities to build strong relationships that can be used by individuals to continuously fulfill, acquire, or create new opportunities for gainful employment. In conclusion, balance means that individuals have the competence to reconcile different elements in the work and/or non-work domains that may be difficult to unite and require fine-tuning. For example, individuals have to continuously balance their current and future work-related (career) goals, their own and their employers’ interests, and their work, career, and non-work interests (Van der Heijde and Van der Heijden, 2006).

### The Relationship Between Age Group Membership and (Self-Perceived) Employability

It can be argued that older workers have gained more work experience and competences over the life-span than their younger counterparts (Froehlich et al., 2015). However, this does not necessarily predict their self-perceived employability to be higher. Building on SST (Carstensen et al., 1999; Carstensen, 2006), older workers can be expected to be less motivated to invest in their employability, since they attach less value to opportunities for advancement and continuous learning (Kooij et al., 2011). SST states that individuals may either have an open or a limited perspective regarding their remaining time at work and their future career opportunities. In the latter case, they focus more on time constraints and reduced opportunities in work and the rest of life (Zacher and De Lange, 2011), which influences their work motivation. Although Carstensen (2006) stresses that an individual’s subjective time horizon should be viewed as a construct that needs to be distinguished from chronological age, the literature generally shows chronological age and future time perspective to be highly correlated (Lang and Carstensen, 2002; Akkermans et al., 2016). When workers have a more limited future time perspective, which is more likely the case for older than for younger workers, they tend to select and set goals that provide emotional well-being and that can be achieved in the shorter run. In a similar vein, it can be argued that when time is perceived to be more open-ended, adjusting to changes in one’s work and occupation, and acquiring up-to-date employability, may be perceived as more rewarding. Therefore, we argue that it is likely that individuals’ focus on employability changes over the life course. Based on this, it can be posited that older workers expect less return on investment in employability enhancement, as they will be closer to their pension age, in comparison with younger workers. Anticipating this, they may be less motivated to maintain and enhance their employability.

### The Moderating Role of Negative (Meta-)Stereotyping Regarding Older Workers in the Relationship Between Older Age Group Membership and (Self-Perceived) Employability

Individuals’ attitudes, motivations, experiences, feelings and behaviors (Kunze et al., 2011; cf. Boehm et al., 2014) regarding investing in employability may be affected by their perceptions of the age-biased climate in their organization, which refers to the process of systematic stereotyping and discrimination against workers merely on the grounds of their age group membership (ibid.). Finkelstein and Farrell (2007) distinguished three age-bias components: (1) the cognitive component, referred to as “stereotyping;” (2) the affective component, referred to as “prejudice;” and (3) the behavioral component, referred to as “discrimination.” Age-based stereotyping, focused on in this study, generally refers to organizational members’ perceptions about the age-related cognitions at the aggregate organizational level. Such perceptions result from interpersonal processes and events between managers and supervisors, coworkers, and, if applicable, clients. On the one hand, older age group members may for example be perceived as less productive, less capable or willing to adopt new technologies, or less committed to organizational change than their younger counterparts (Henkens, 2005). On the other hand, older age group members may be perceived as more experienced, more loyal to their organization, or more trustworthy (Henkens, 2005).

Building upon self-categorization theory (Turner, 1982; Tajfel and Turner, 1986), we posit that older workers themselves may be aware of, or may perceive negative stereotypes regarding their age group (e.g., from their managers, coworkers, or clients; see also Crocker et al., 1998). In fact, older workers may have formed their own opinion about how the age groups they belong to is perceived by outgroup members (Sigelman and Tuch, 1997) and may even identify with the perceptions they hold of outgroup members’ beliefs toward them. The beliefs regarding the stereotypes that outgroup members may hold about them can be referred to as meta-stereotypes, which often have a negative character (Vorauer et al., 1998), and can, therefore, reduce the degree of self-worth of the stereotyped group (Owuamalam and Zagefka, 2011, 2014). Importantly, however, meta-stereotyped views held by ingroup members do not have to be in line with the actual beliefs that the outgroup members hold of them (cf. Finkelstein et al., 2013). Yet, they determine how individuals view the world (Owuamalam and Zagefka, 2013).

When older workers perceive that their age group is negatively stereotyped, they may experience anxiety or anger (King et al., 2008), which in turn may influence their work outcomes (Ryan et al., 2015). In such situations, older workers may find it difficult to hold positive views about themselves, possibly affecting their self-perceived employability. Consequently, the older workers may also perceive that they have less opportunities and more limitations to develop their employability (cf. Froehlich et al., 2015), which can become a *self-fulfilling prophecy* (Merton, 1948). Hence, when older workers engage in meta-stereotyping, it can both affect their self-perceived employability, and can reduce their motivation to further invest in developing or maintaining their employability, herewith creating a vicious circle (cf. Branscombe et al., 1999; Schmitt et al., 2002; Dordoni et al., 2017).

**FIGURE 1:**
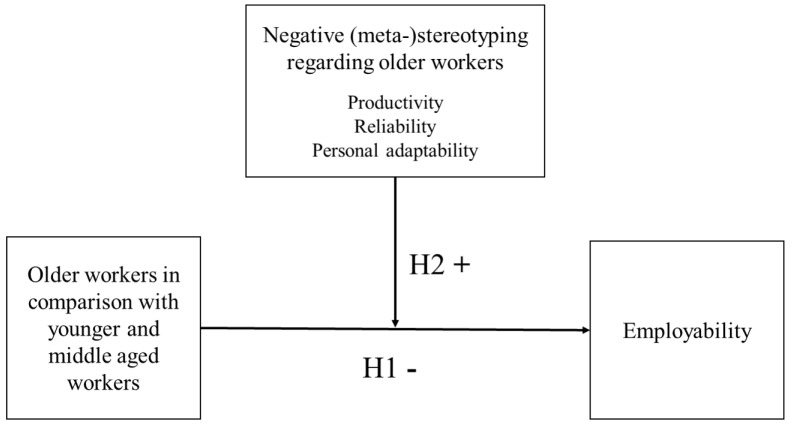
Conceptual model presenting the moderating role of negative (meta-)stereotyping regarding older workers in the relationship between older age group membership and self-perceived employability.

Based on the psychological mechanisms presented above, we propose the following (see also Figure 1):

**Hypothesis 1**: Older workers report lower levels of employability than their younger counterparts.
**Hypothesis 2**: Negative (meta-)stereotyping regarding older workers’ productivity, reliability and personal adaptability amplifies the negative effect of older workers’ age on their self-perceived employability.

## Materials and Methods

### Sample and Procedure

Data (*N* = 98) were gathered in seven branches of a large Dutch supermarket chain employing 1,240 shop-floor workers. Our contact person, supporting the field work, indicated that in view of the contextual developments mentioned in the Introduction, HRM aimed to improve the position of older age group members in the organization by enhancing their employability. In the present study, we only included workers meeting our criterion of having a permanent employment contract for at least 12 h per week as it is more likely that these workers have insight into the organizational climate. The response rate was 26.34%. Participants’ mean age was 34.78 years (*SD* = 13.25). They were contracted, on average, for 83 h on a monthly basis (*SD* = 53.61), and had been employed, on average, for 14.81 years (*SD* = 10.93). About half of them (53%) had enjoyed higher education. The sample consisted of slightly more men (58%) than women.

### Age Group Membership

Age-group membership was measured by a set of dummy variables: the younger age group (< 30 years old, *n* = 47); the middle-aged group (30 to 49 years old, *n* = 32); and the older age group (being the reference category in our empirical analysis) (50 to 67 years old, *n* = 19). This age group categorization is commonly used in the literature. However, in view of our focus on low-skilled job incumbents who can start their careers at a relatively young age, the threshold between what we consider younger versus middle aged worker categories (i.e., 30 years) is slightly lower than in other studies (i.e., 35 years) (cf. Van der Heijden, 2001).

### Self-Perceived Employability

We used Van der Heijde and Van der Heijden’s (2006) validated self-perceived employability scale, comprising five competence-based dimensions: Occupational Expertise (e.g., *During the past year*, *I was*, *in general*, *competent to perform my work accurately and with few mistakes*); Anticipation and Optimization (e.g., *I take responsibility for maintaining my labor market value*); Corporate Sense (e.g., *I support the operational processes within my organization*); Personal Flexibility (e.g., *I adapt to developments within my organization*); and Balance (e.g., *My work and private life are evenly balanced*). The Cronbach’s alphas of the subscales ranged from 0.62 (personal flexibility) to 0.87 (occupational expertise and balance) (see also Table 1).

**Table 1:** Means, standard deviations, and pearson’s correlations between study variables.

	*M*	*SD*	1	2	3	4	5	6	7	8	9	10	11	12
(1) Chronological age	34.78	13.25												
(2) Gender	–	–	0.04											
(3) Organizational tenure	14.81	10.93	0.81***	-0.04										
(4) Contractual work hours	83.33	53.61	0.51***	-0.31**	0.51***									
(5) Education level	–	–	-0.48***	-0.09	-0.43***	-0.18								
(6) MST Productivity	2.71	0.75	-0.21*	-0.04	-0.20*	-0.12	0.07							
(7) MST Reliability	2.80	0.73	-0.17*	0.18	-0.18	-0.16	0.14	-0.07						
(8) MST Adaptability	3.22	0.66	-0.12	-0.05	-0.16	-0.03	0.17	0.36***	-0.25*					
(9) Occupational expertise	4.88	0.42	-0.56*	-0.03	-0.15	-0.06	0.12	0.21*	0.03	0.16				
(10) Anticipation and optimization	4.05	0.69	-0.19	-0.05	-0.08	0.01	-0.08	-0.01	0.09	0.03	0.29**			
(11) Corporate sense	4.41	0.70	-0.15	-0.18	-0.02	0.15	-0.02	0.12	0.08	0.11	0.49***	0.67***		
(12) Personal Flexibility	4.50	0.43	-0.28*	-0.04	-0.30**	-0.22*	0.12	0.18	0.24*	0.10	0.48***	0.33**	0.42***	
(13) Balance	4.42	0.70	-0.11	0.01	-0.05	-0.14	-0.16	-0.03	0.01	-0.13	0.31**	0.43***	0.33**	0.14


*N = 98, MST = Perceived age-based (meta-)stereotyping, ^∗^p < 0.05, ^∗∗^p < 0.01, ^∗∗∗^p < 0.001.*

### Perceived Negative (Meta-)Stereotyping in the Organization Regarding Older Age Group Members

To measure respondents’ perceptions of age-based (meta-)stereotyping in their organization (in this study operationalized as what the respondent perceives that organizational members think about older workers), we adjusted the three-dimensional validated instrument by Henkens (2005), based on the 15 original items measuring three stereotyping dimensions: Productivity (e.g., *Older workers are less productive than younger workers*); Reliability (e.g., *Older workers are more reliable than younger workers*); and Personal Adaptability (e.g., *Older workers are less interested in technological change than younger workers*). For example, the item “Older workers are less productive than younger workers” was rephrased as follows: “In my organization, it is believed that older workers are less productive than younger workers.” Perceptions of stereotyping regarding older age group members were coded such that higher values on the three dimensions represented respondents to perceive more *negative* (meta-)stereotypes regarding older workers’ productivity, reliability and personal adaptability, respectively. The Cronbach’s alphas were as follows: 0.71 (productivity), 0.79 (reliability), and 0.70 (personal adaptability). Since our instrument measures “perceived stereotyping regarding older age group members in the organization,” the responses of the older workers can be regarded to measure meta-stereotyping.

### Analyses

We tested our hypotheses, depicted in Figure 1, by conducting five different series of moderated hierarchical regression analyses, i.e., one for each of the five distinguished employability dimensions. All predictor variables were standardized for a better interpretation of the results. First, we included the study’s control variables (i.e., gender, organizational tenure, contractual work hours, and educational level), because these variables can be assumed to be related to employability and/or age in general (Spector and Brannick, 2011). Second, we included the age group membership dummy variables. Third, we added the three age-based (meta-)stereotypes’ variables (regarding older workers’ productivity, reliability, and personal adaptability). Hypothesis 1 was tested based on the results of step three. In a fourth step, we added and tested the interactions between the age group membership dummies and age-based (meta-)stereotypes. Hypothesis 2 was tested based on the results of step four. In case of significant interactions, we plotted the interaction to provide a better understanding of its meaning. As we formulated directional hypotheses and because of difficulties for detecting interaction effects with a low sample size (Cohen et al., 2013), we applied one-sided significance tests in the regression analyses (Cho and Abe, 2013). Table 1 presents the means, standard deviations and correlations between the study variables. Table 2 presents the results of the moderated regression analyses.

**Table 2:** Moderated regression analyses for perceived age-based stereotyping and age-group as predictor of five employability dimensions.

	Occupational expertise			Anticipation and optimization			Corporate sense			Flexibility			Balance		
Variable	*B*	*SE*	β	*B*	*SE*	β	*B*	*SE*	β	*B*	*SE*	β	*B*	*SE*	β
*Step 1*															
Gender	-0.01	0.09	-0.02	0.00	0.16	0.00	-0.19	0.16	-0.13	-0.05	0.10	-0.06	-0.09	0.16	-0.07
Organizational tenure	-0.06	0.05	-0.15	-0.14	0.09	-0.20	-0.10	0.09	-0.14	-0.11	0.06	-0.26*	-0.07	0.09	-0.10
Contractual work hours	0.03	0.05	0.06	0.07	0.09	0.10	0.13	0.09	0.19	-0.04	0.06	-0.10	-0.09	0.09	-0.12
Education level	0.02	0.10	0.02	-0.20	0.16	-0.14	-0.10	0.16	-0.07	-0.02	0.10	-0.02	-0.39	0.16	-0.28**
Change in *R^2^*	0.02			0.03			0.06			0.10*			0.08†		
*Step 2*															
Gender	0.02	0.10	0.02	0.12	0.16	0.01	-0.07	0.16	-0.05	-0.04	0.10	-0.05	-0.01	0.16	0.00
Organizational tenure	0.02	0.07	0.04	0.02	0.12	0.03	0.14	0.12	0.19	-0.07	0.08	-0.15	0.08	0.13	0.11
Contractual work hours	0.04	0.06	0.09	0.17	0.10	0.24*	0.19	0.10	0.27*	-0.06	0.06	-0.14	-0.03	0.10	-0.04
Education level	0.00	0.10	0.00	-0.31	0.17	-0.22*	-0.18	0.17	-0.13	0.00	0.10	0.00	-0.47	0.17	-0.33**
Young workers (ref. = older workers)	0.28	0.20	0.35	0.70	0.34	0.51*	0.92	0.33	0.65**	0.15	0.21	0.17	0.60	0.34	0.42*
Middle-aged workers (ref. = older workers)	0.21	0.15	0.25	0.18	0.25	0.12	0.58	0.25	0.38*	0.22	0.15	0.24	0.26	0.25	0.17
Change in *R^2^*	0.03			0.06*			0.08*			0.02			0.03		
*Step 3*															
Gender	0.01	0.10	0.02	0.09	0.17	0.06	-0.11	0.16	-0.08	-0.07	0.10	-0.08	0.00	0.17	0.00
Organizational tenure	0.03	0.08	0.07	0.04	0.13	0.05	0.17	0.12	0.25	-0.04	0.08	-0.09	0.06	0.13	0.08
Contractual work hours	0.04	0.06	0.09	0.17	0.10	0.24	0.19	0.10	0.27*	-0.05	0.06	-0.12	-0.02	0.10	-0.03
Education level	-0.01	0.10	-0.01	-0.33	0.17	-0.24*	-0.23	0.17	-0.16	-0.03	0.10	-0.03	-0.45	0.17	-0.32**
Young workers (ref. = older workers)	0.27	0.20	0.33	0.72	0.34	0.52*	0.93	0.33	0.66**	0.15	0.20	0.18	0.63	0.34	0.44*
Middle-aged workers (ref. = older workers)	0.18	0.15	0.22	0.19	0.26	0.13	0.56	0.25	0.37*	0.21	0.15	0.22	0.31	0.26	0.21
MST Productivity	0.04	0.05	0.10	-0.01	0.08	-0.01	0.03	0.08	0.04	0.04	0.05	0.09	-0.04	0.08	-0.06
MST Reliability	0.03	0.05	0.07	0.09	0.08	0.13	0.14	0.08	0.20*	0.11	0.05	0.25*	-0.01	0.08	-0.01
MST Adaptability	0.03	0.05	0.07	0.02	0.08	0.02	0.08	0.08	0.11	0.03	0.05	0.06	-0.09	0.08	-0.13
Change in *R^2^*	0.02			0.02			0.04			0.06			0.02		
*Step 4*															
Gender	-0.01	0.10	-0.02	0.13	0.16	0.09	-0.13	0.17	-0.09	-0.11	0.11	-0.12	0.15	0.17	0.11
Organizational tenure	0.04	0.08	0.09	0.02	0.12	0.03	0.18	0.12	0.25	-0.03	0.08	-0.08	-0.02	0.12	-0.02
Contractual work hours	0.02	0.06	0.04	0.13	0.10	0.19	0.15	0.10	0.21	-0.06	0.06	-0.15	-0.02	0.10	-0.02
Education level	-0.02	0.11	-0.03	-0.31	0.17	-0.22*	-0.25	0.18	-0.18	-0.03	0.12	-0.03	-0.46	0.18	-0.33**
Young workers (ref. = older workers)	0.41	0.21	0.52*	0.95	0.33	0.68**	10.13	0.34	0.80**	0.16	0.22	0.18	0.70	0.34	0.50*
Middle-aged workers (ref. = older workers)	0.36	0.16	0.43*	0.52	0.26	0.35*	0.85	0.27	0.56**	0.25	0.17	0.27	0.44	0.26	0.29
MST Productivity	-0.10	0.09	-0.25	-0.31	0.15	-0.45*	-0.14	0.15	-0.19	0.08	0.10	0.17	-0.31	0.15	-0.44*
MST Reliability	-0.11	0.09	-0.29	-0.20	0.15	-0.29	-0.11	0.15	-0.15	0.02	0.10	0.05	0.01	0.15	0.02
MST Adaptability	-0.14	0.10	-0.34	-0.39	0.16	-0.57**	-0.29	0.17	-0.41*	-0.05	0.11	-0.11	-0.35	0.17	-0.49*
Young E × MSTP	0.21	0.12	0.35*	0.47	0.19	0.45**	0.27	0.19	0.25	-0.04	0.12	-0.07	0.39	0.19	0.36*
Young E × MSTR	0.17	0.12	0.28	0.51	0.18	0.49**	0.35	0.19	0.34*	0.09	0.12	0.14	0.13	0.19	0.13
Young E × MSTA	0.18	0.13	0.31	0.43	0.20	0.42*	0.41	0.21	0.40*	0.13	0.13	0.20	0.37	0.21	0.36*
Middle-aged E × MSTP	0.09	0.13	0.12	0.20	0.21	0.16	0.00	0.21	0.00	-0.12	0.14	-0.15	0.31	0.21	0.24
Middle-aged E × MSTR	0.23	0.13	0.31*	0.13	0.20	0.10	0.27	0.21	0.20	0.18	0.13	0.22	-0.42	0.21	-0.31*
Middle-aged E × MSTA	0.22	0.14	0.31	0.51	0.23	0.40*	0.52	0.24	0.41*	0.08	0.15	0.10	0.07	0.23	0.06
Change in *R^2^*	0.09			0.19**			0.10†			0.04			0.17**		


*N = 98, ST = age-based (meta-)stereotyping, MSTP = age-based (meta-)stereotyping productivity, MSTR = age-based (meta-)stereotyping reliability, MSTA = age-based (meta-)stereotyping adaptability, ^†^p < 0.10, ^∗^p < 0.05, ^∗∗^p < 0.01, ^∗∗∗^p < 0.001.*

## Results

### Bivariate Analysis

We first analyzed the correlation matrix (see Table 1), which reveals that the correlations between the variables chronological age and two age-based (meta-)stereotypes are negative: older workers’ productivity (*r* = -0.21, *p* < 0.05); and reliability (*r* = -0.17, *p* < 0.05). These correlations reveal that perceptions of negative age-based stereotyping are larger among the two younger age groups than among the older age group. However, chronological age was not significantly related to age-based (meta-)stereotypes regarding older workers’ personal adaptability (*r* = -0.12, *ns*).

### Hypotheses Testing

#### Occupational Expertise

**FIGURE 2:**
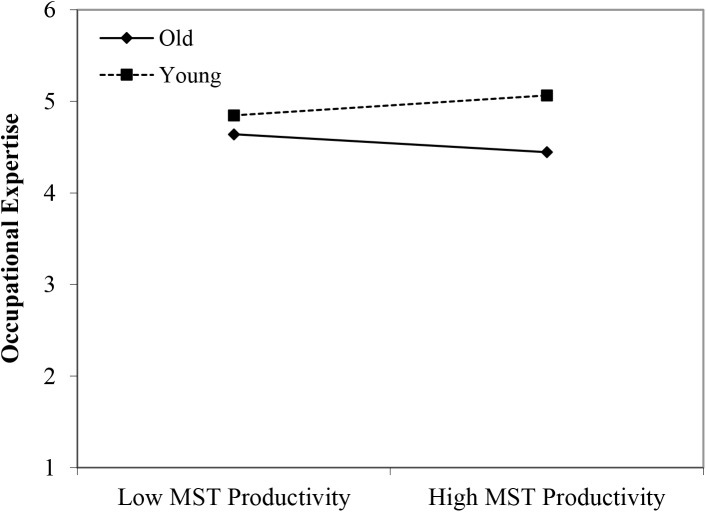
Interaction between the age-based (meta-)stereotyping of productivity and age groups: Predicting occupational expertise. Low = –1 *SD*, High = + 1 *SD*, MST = age-based (meta-)stereotyping, old = older workers, young = younger workers.

Contrary to Hypothesis 1, we found no main effect of age group membership for perceived occupational expertise. Also no main effect of perceived (meta-)stereotyping regarding older workers was found. However, partly in line with Hypothesis 2, we found two significant interactions: young workers × age-based stereotypes on *productivity* (β = 0.35, *p* < 0.05); and middle-aged workers × age-based stereotype *reliability* (β = 0.31, *p* < 0.05). An inspection of the plots in Figure 2, 3 shows that self-perceived occupational expertise was more negatively affected by age group membership (older compared to younger and middle-aged workers) under conditions of stronger perceptions of (meta-)stereotyping regarding older workers’ productivity and reliability, respectively. These findings can be taken to indicate effects of older workers engaging in meta-stereotyping.

**FIGURE 3:**
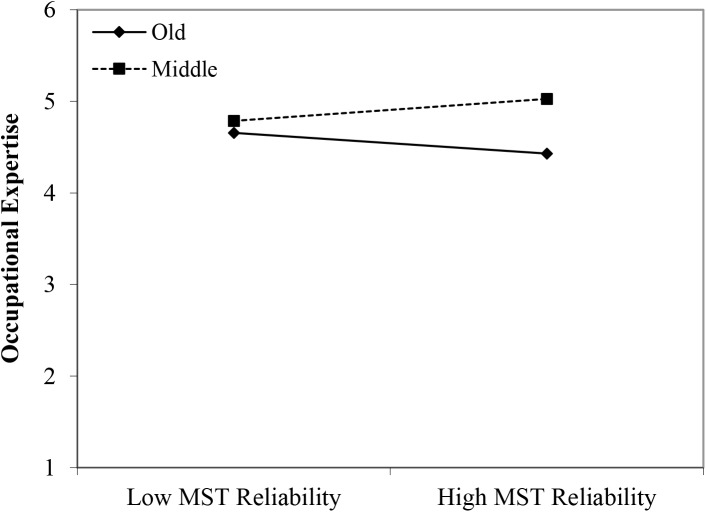
Interaction between age-based (meta-)stereotyping of reliability and age groups: Predicting occupational expertise. Low = –1 *SD*, High = + 1 *SD;* ST = age-based (meta-)stereotyping old = older workers; middle = middle-aged workers.

#### Anticipation and Optimization

**FIGURE 4:**
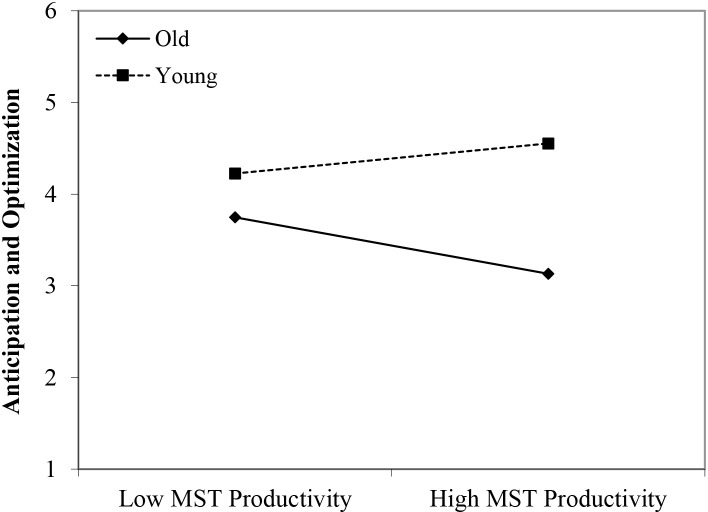
Interaction between perceived age-based (meta-)stereotyping productivity and age groups: Predicting anticipation and optimization. Low = –1 *SD*, High = + 1 *SD*, MST = perceived age-based (meta-)stereotyping, old = older workers, young = younger workers.

**FIGURE 5:**
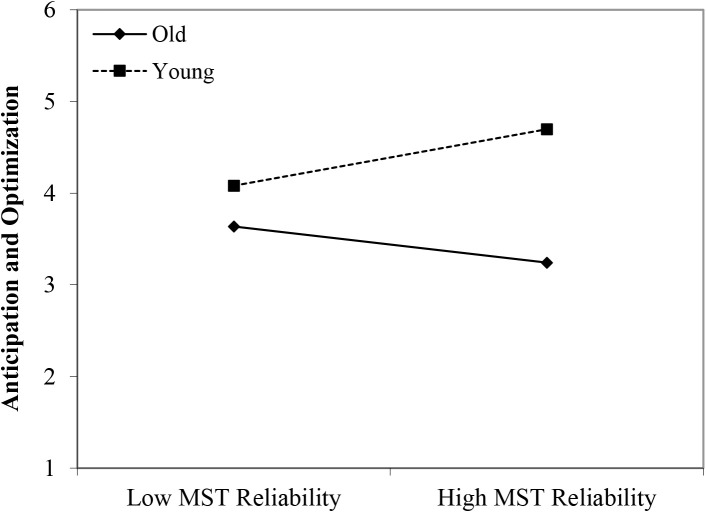
Interaction between perceived age-based (meta-)stereotyping reliability and age groups: Predicting anticipation and optimization. Low = –1 *SD*, High = + 1 *SD*, MST = perceived age-based (meta-)stereotyping, old = older workers, young = younger workers.

**FIGURE 6:**
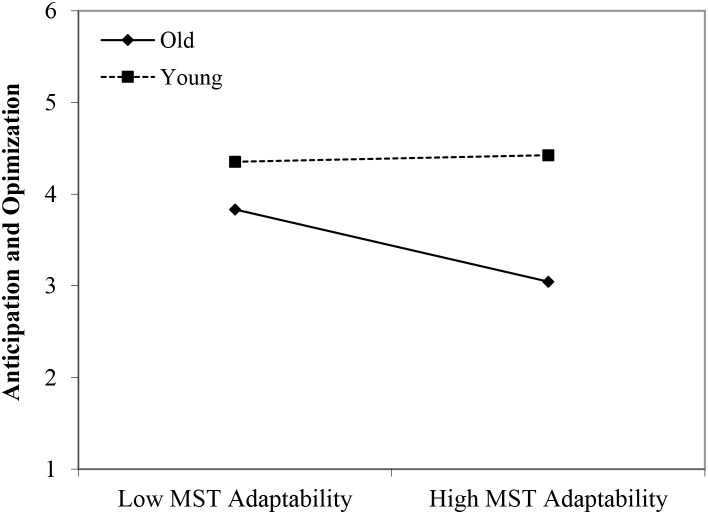
Interaction between perceived age-based (meta-)stereotyping adaptability and age groups: Predicting anticipation and optimization. Low = –1 *SD*, High = + 1 *SD*, MST = age-based (meta-)stereotyping, old = older workers, young = younger workers.

**FIGURE 7:**
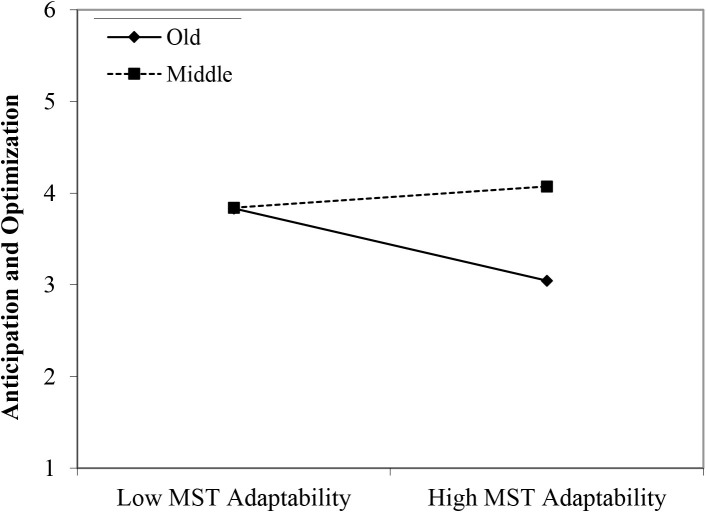
Interaction between perceived age-based (meta-)stereotyping adaptability and age groups: Predicting anticipation and optimization. Low = –1 *SD*, High = + 1 *SD*, MST = age-based (meta)stereotyping, old = older workers, middle = middle-aged workers.

In line with Hypothesis 1, we found that younger workers scored higher on anticipation and optimization than older workers (β = 0.52, *p* < 0.05). Although we did not find any main effects of perceived negative age-based (meta-)stereotyping, four significant interactions could be identified, partly confirming Hypothesis 2: young workers × age-based stereotypes on productivity (β = 0.45, *p* < 0.01); young workers × age-based stereotypes on reliability (β = 0.49, *p* < 0.001); young workers × age-based stereotypes on personal adaptability (β = 0.42, *p* < 0.05); and middle-aged workers × age-based stereotypes on personal adaptability (β = 0.40, *p* < 0.05). In every case, workers’ self-ratings of anticipation and optimization were more strongly negatively affected by age group membership (older age group compared to younger and middle-aged groups) under conditions of stronger perceptions of negative stereotypes regarding older workers (see Figure 4–7). These findings can be taken to indicate effects of older workers engaging in meta-stereotyping.

#### Corporate Sense

**FIGURE 8:**
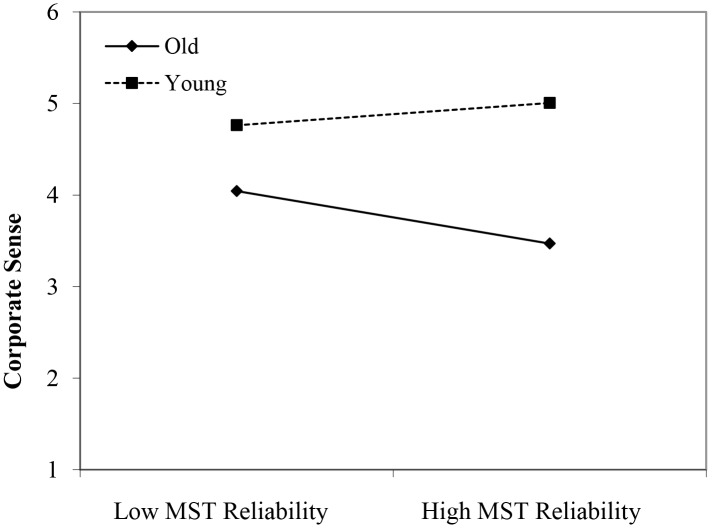
Interaction between perceived age-based (meta-)stereotyping reliability and age groups: Predicting corporate sense. Low = -1 *SD*, High = + 1 *SD*, MST = age-based (meta-)stereotyping reliability, old = older workers, young = younger workers.

**FIGURE 9:**
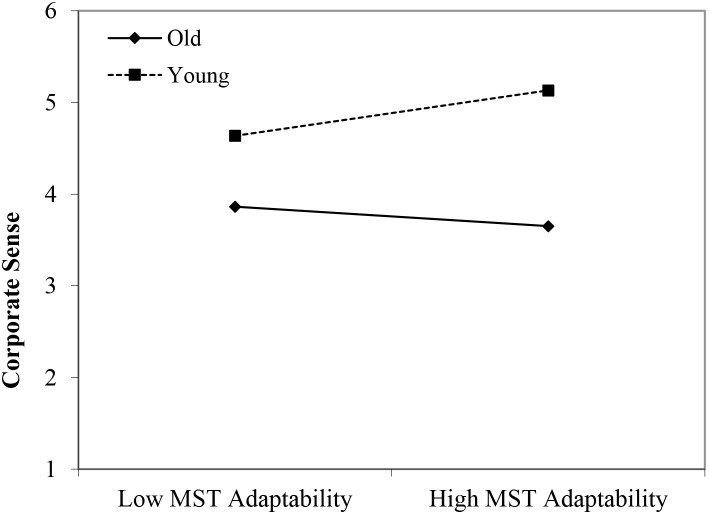
Interaction between perceived age-based (meta-)stereotyping adaptability and age groups: Predicting corporate sense. Low = –1 *SD*, High = + 1 *SD*, MST = age-based (meta-)stereotyping, old = older workers, young = younger workers.

**FIGURE 10:**
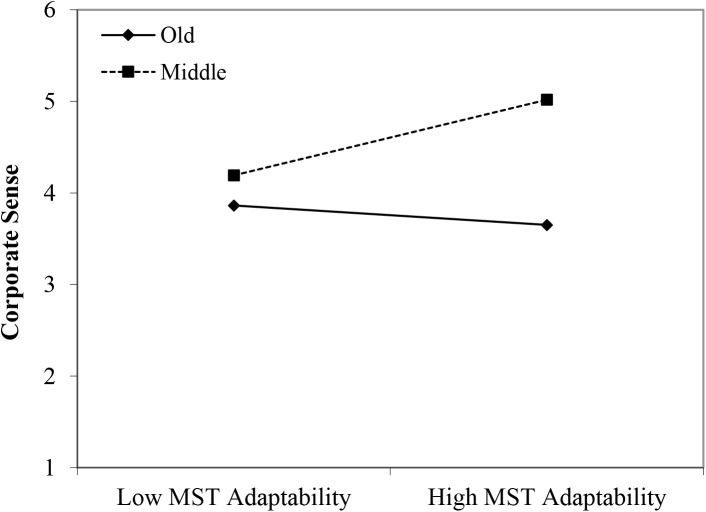
Interaction between perceived age-based (meta-)stereotyping adaptability and age groups: Predicting corporate sense. Low = –1 *SD*, High = + 1 SD, MST = perceived age-based (meta-)stereotyping adaptability, old = older workers, middle = middle-aged workers.

In line with Hypothesis 1, both the younger age group (β = 0.66, *p* < 0.001) and the middle-aged group (β = 0.37, *p* < 0.05) reported higher scores on corporate sense than their older age counterparts. Moreover, the perceived stereotypes regarding older workers’ reliability appeared to have a positive main effect (β = 0.20, *p* < 0.05). In line with Hypothesis 2, we found three significant interactions between age group membership and perceived age-based (meta-)stereotyping: young workers × age-based stereotypes on reliability (β = 0.34, *p* < 0.005); young workers × age-based stereotypes on personal adaptability (β = 0.40, *p* < 0.05); and middle-aged workers × age-based stereotypes on personal adaptability (β = 0.41, *p* < 0.05). Again, in the case of stronger perceptions of stereotyping regarding older workers, the data revealed that particularly in the older age group, a negative relationship between (meta-)stereotyping and corporate sense emerged (see Figure 8–10). These findings can be taken to indicate effects of older workers engaging in meta-stereotyping.

#### Personal Flexibility

No significant main effect of age group membership on personal flexibility was found and only one positive main effect of age-based stereotyping was identified: stereotypes on reliability (β = 0.25, *p* < 0.05). With this outcome, Hypotheses 1 and 2 were not supported with our data.

#### Balance

**FIGURE 11:**
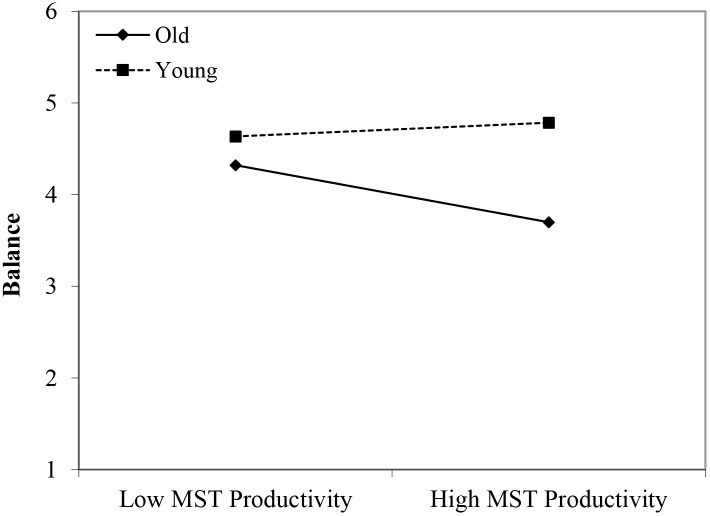
Interaction between perceived age-based (meta-)stereotyping productivity and age groups: Predicting balance. Low = –1 *SD*, High = + 1 *SD*, MST = perceived age-based (meta-)stereotyping, old = older workers, young = younger workers.

**FIGURE 12:**
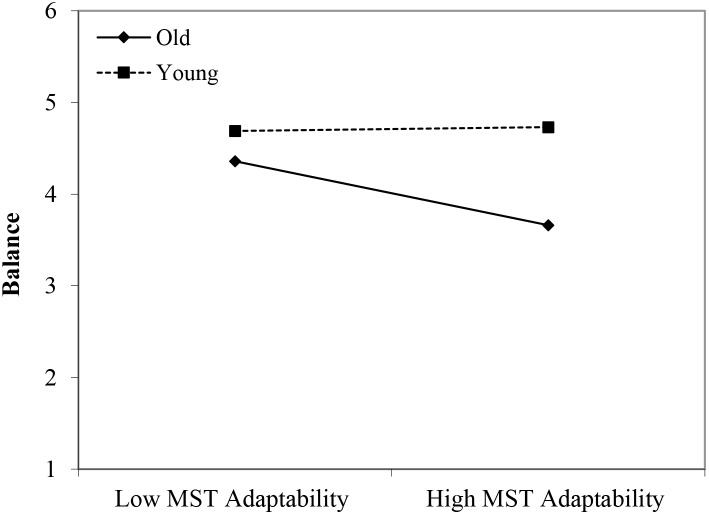
Interaction between perceived age-based (meta-)stereotyping adaptability and age groups: Predicting balance. Low = –1 *SD*, High = + 1 *SD*, MST = perceived age-based (meta-)stereotyping adaptability, old = older workers, young = younger workers.

**FIGURE 13:**
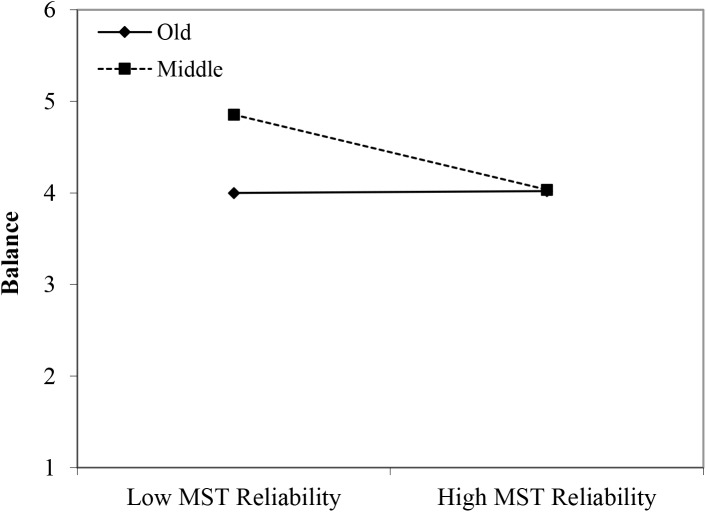
Interaction between perceived age-based (meta-)stereotyping reliability and age groups: Predicting balance. Low = –1 *SD*, High = + 1 *SD*, MST = perceived age-based (meta-)stereotyping, old = older workers, middle = middle-aged workers.

In line with Hypothesis 1, the younger workers reported more balance in comparison with the older group (β = 0.44, *p* < 0.05). Although no significant main effects of perceived age-based stereotyping were identified, we found significant interactions for all three types of age-based stereotyping: young workers × age-based stereotypes on productivity (β = 0.36, *p* < 0.05); young workers × age-based stereotypes on adaptability (β = 0.36, *p* < 0.05); and middle-aged workers × age-based stereotypes on reliability (β = -0.31, *p* < 0.05). Regarding the younger age group, the findings were in line with Hypothesis 2. That is, under conditions of stronger perceptions of age-based stereotyping (i.e., regarding productivity and adaptability), stronger negative effects were found for the older age group in comparison with the younger age group (see Figure 11, 12). However, the interaction effect between the middle-aged group and stereotyping regarding older workers’ reliability showed an unexpected direction (see Figure 13). The data revealed that it was the middle-aged group that reported a stronger negative relationship between the age-based stereotype of reliability and balance in comparison with older workers. These findings can be taken to indicate effects of both middle aged workers engaging in stereotyping and older workers engaging in meta-stereotyping.

## Discussion and Conclusion

Nowadays, employers and managers increasingly realize that supporting workers’ employability enhancement through SCD policies and practices is important (De Vos et al., 2016). Despite this, however, organizations still tend to focus on high potentials only, rather than taking an inclusive approach (De Vos and Dries, 2013; Peters and Lam, 2015). Consequently, more disadvantaged groups in the workforce (e.g., older workers, women workers, immigrants and less qualified workers) may be excluded from organizations’ employability policies and practices. As a consequence, members of these groups may refrain from investing in their own employability, such as training, which may create a self-fulfilling prophecy (Merton, 1948; Dordoni et al., 2017). Stereotypes held by group members about themselves might affect this process. Therefore, in this empirical study, we addressed the role of age group membership and negative age-related (meta-)stereotyping regarding older workers, and, particularly, the amplifying role the latter may have for (older) workers’ self-perceived employability.

### Summary and Reflection

First, our descriptive analyses (presented in Table 1) showed that older age group members perceive less negative stereotyping in their organization regarding older workers’ productivity and reliability (but not regarding personal adaptability) than the younger age group members. Possibly, the younger age groups perceive more negative age-based stereotyping because they are dissimilar to their older co-workers, for example as regards their physical appearance and values/interests (Ryan et al., 2015). In a related vein, older workers may experience less negative age-based stereotyping about themselves (i.e., meta-stereotypes) as they can more easily distance themselves from negative age stereotyping (e.g., “I am not that old”) (ibid.).

Second, based on our explanatory analyses (see the direct effects of age group membership presented in Table 2), overall, and contrary to our expectations, we found that older workers did not report lower levels of *occupational expertise* and *personal flexibility*. A possible explanation may be that the type of work conducted by the supermarket workers focused on in this study does not demand that much education and mainly comprises routine-based service work, perhaps characterized by relatively low levels of emotional, physical and cognitive demands. This may be different for workers in other industries, such as workers in high manual and physically demanding jobs. For example, warehouse workers may face stronger physical demands due to lean production processes (Peters and Lam, 2015), whereas knowledge workers in high tech organizations operating in turbulent markets, may experience knowledge, tools and techniques to be constantly in flux. Possibly, the older workers in our study may not have experienced such rapidly changing demands in their jobs and may therefore feel that they are able to keep up with occurring changes in the workplace.

In line with expectations based on SST (Carstensen et al., 1999; Carstensen, 2006), members of the oldest age group in our study reported lower levels of *anticipation and optimization* in comparison with the youngest age group (but not with the middle-aged group). Due to their more limited life-time horizon regarding work (and non-work), older workers may hold a less proactive attitude toward searching for other career possibilities in- or outside the organization than their younger counterparts who have a more open-ended time horizon (cf. Carstensen, 2006).

Older age group members also reported less *corporate sense* in comparison with members from the two younger age groups. Building upon SST, it might be that given their increased focus on non-work goals in view of their more limited future time perspective, older workers may be less eager to display corporate sense, such as team and organizational commitment, since they are less motivated to create new opportunities for gainful employment, for example by sharing knowledge at team and organizational levels and by investing in solid collaborations at work. This may especially be the case when work is less challenging (cf. Petrou et al., 2017). Presumably, the lower levels of corporate sense may decrease older workers’ social capital and, consequently, their odds of finding (new) employment if necessary.

Finally, older workers generally reported lower *balance*, but only in comparison with the youngest age group. In view of their more limited future time perspective, it is conceivable that older workers might prioritize non-work goals more than younger workers. According to SST, in this stage of their working life, achieving non-work goals may provide older workers more emotional well-being than achieving work goals. Alternatively, older workers may have more non-work obligations, such as informal care for elderly, which demands them to shift their attention to the non-work domain. Yet, enhancing older workers’ employability in this regard would also allow them to develop what can be referred to a “protean career” based on personal values (Hall and Mirvis, 1995) which may be both emotionally and financially rewarding.

Third, also based on our explanatory analyses (see the interaction effects between age and negative stereotypes presented in Table 2), we found that those older workers who did perceive more negative stereotypes regarding their own age group (which can be indicated as those older workers who engaged in more negative meta-stereotyping) indeed reported lower scores on some of the employability dimensions than others who perceived similar negative age-based stereotypes. In fact, most of the interactions between age group and stereotypes regarding older workers revealed differences between older versus younger workers. However, also differences between middle-aged and older workers were found.

In comparison with workers in the youngest age group holding similar perceptions, older workers who perceived more negative stereotyping regarding older workers’ productivity reported lower levels of *occupational expertise*. In comparison with the middle-aged group, older workers who experienced more negative stereotyping regarding older workers’ reliability reported lower levels of *occupational expertise*. In line with our theoretical framework, it can be argued that meta-stereotyping undermines older workers’ sense of self-worth and reduces their opportunity focus (Froehlich et al., 2015). Consequently, they may become demotivated to further invest in their employability enhancement (cf. Branscombe et al., 1999; Schmitt et al., 2002; Dordoni et al., 2017), which is reflected in lower self-evaluations.

In a related vein, in comparison with younger workers, older workers’ self-perceived *anticipation and optimization* was negatively affected by negative age-based meta-stereotyping on productivity, reliability, and personal adaptability. Perceived stereotyping of older workers’ personal adaptability was also associated with less *anticipation and optimization* of older workers in comparison with middle-aged workers. Building upon our theoretical framework, we can argue that older workers’ meta-stereotyping may lead to feelings of uncertainty, demotivating them more strongly than others to display proactive labor market behavior.

The negative (meta-)stereotyping regarding older workers’ reliability was only associated with lower self-perceptions of *corporate sense* among older in comparison with younger workers. Moreover, older workers who did perceive more negative stereotyping regarding their personal adaptability scored lower on *corporate sense* compared with both younger age-groups. Possibly, older workers who experience stronger negative stereotyping regarding their own age group are less inclined to invest in high-quality relationships and interactions with other members in the team and organization as this may not pay-off. For *personal flexibility*, no significant interaction effects between age group and age-based (meta-)stereotypes were found.

Especially in comparison with the youngest age group, older workers’ negative meta-stereotyping (associated with their productivity and personal adaptability) was associated with a lower view on *balance* between own work and non-work objectives and between personal and employer goals. Following SST, this finding may reflect that older workers are inclined to pursue more non-work goals which they have to balance with their work obligations. In light of perceived negative meta-stereotypes, older workers might not expect many opportunities in and rewards from their work and disengage from their work goals, or perhaps develop a higher engagement in non-work goals (cf. Petrou et al., 2017), which makes it particularly difficult for them to perceive balance between work and non-work. Strikingly, however, the gap between the older workers’ and middle aged workers’ reported *balance* is less wide when both perceive negative stereotyping regarding older workers’ reliability. Possibly, when older workers feel that they are not perceived as reliable, they compensate for this by gaining a better balance. Alternatively, when the middle aged workers perceive older workers to be less reliable, they may feel that they have to substitute for others, or cope with emergencies more than older workers, affecting their perceived balance.

### Limitations and Future Research

Our study provides evidence on the role of psychological processes associated with age group membership and its interaction with age-based (meta-)stereotyping in the light of self-perceived employability. Future research is needed to cross-validate our findings, especially given our relatively small sample size, the self-report and cross-sectional nature of our data, and our focus on workers in the supermarket/service industries.

In our study, we addressed an under-studied population of workers when it comes to career development, i.e., older less qualified (supermarket) service workers. It would be valuable, however, to focus future research on older workers in other industries as well, as the relationships between age group membership, (meta-)stereotyping, and individuals’ self-perceived employability, and its dimensions distinguished in this study, may vary across job incumbents with different levels of emotional, physical and cognitive demands. For example, the relationship between age group membership and self-perceived occupational expertise and personal flexibility may depend on the degree of cognitive demands characterizing the type of work and the market developments that pressure workers to keep up with ongoing changes regarding knowledge, techniques, and tools. It is not clear yet, however, how the more limited future time perspective associated with older workers (Carstensen et al., 1999; Carstensen, 2006) affects knowledge workers’ motivation to invest in occupational expertise and to display personal flexibility differently than the supermarket workers in our study, and how these relationships may be contingent upon their perceptions of negative age-based stereotyping in their organization. Older knowledge workers who may experience a more limited life-time horizon regarding work (and non-work) than younger knowledge workers may also be more inclined to display higher levels of *anticipation and optimization* than the supermarket workers in our study. Possibly, knowledge workers face more alternative career possibilities in- or outside their organization, which may also be enabled by their employers’ age-aware (training and demotion) policies (cf. Fleischmann et al., 2015). These are all interesting avenues for future research.

Future research could also address other types of disadvantaged groups and may further investigate the interactions between structural (e.g., job type) and individual factors, such as age, sex and ethnic background, in order to explain how (meta-)stereotyping regarding these groups can impact SDC. In fact, taking into account the future of work, current (labor) market trends (such as labor market shortages) may pressure organizations to look beyond professional and managerial workers regarding career development opportunities (Lawrence et al., 2015) and to also include other groups of workers. Unfortunately, at present, those most in need of reskilling and upskilling still appear to receive far less training than others (World Economic Forum, 2018), possibly leading to meta-stereotyping and self-fulfilling prophecies. However, sustained employability for all will be important, not only for the individuals’ own economic, social and mental sustainability (De Vos and Van der Heijden, 2015), but also for organizational adaptability and sustainable growth. In that regard, future research could focus on possible constraints that, overtly or implicitly, affect how workers are looked at and how they look at themselves and how to overcome (meta-)stereotyping in order to encourage (later) life employment.

### Policy Implications

Our findings also have implications for policy makers and other organizational stakeholders. First, older workers might not be aware of (meta-)stereotyping, but may unwittingly experience its impact. However, our study showed that those who do experience (meta-)stereotyping are likely to suffer from negative effects on their own perceived employability. This suggests that SCD practices need to pay particular attention to the possibly amplifying psychological processes that demotivate older workers to recognize and invest in their own employability, as this may impact their potential to develop a career over the full life cycle that may be both meaningful (Hall and Mirvis, 1995) and that enhances their financial self-sufficiency. In this study, not only processes associated with age group membership in itself, but also the interaction with processes associated with older workers’ meta-stereotyping and other-age groups’ age-based stereotyping were shown to affect workers’ self-perceived employability, and hence their career development opportunities. In order to avoid labor market exclusion of groups that are either actually negatively stereotyped and/or perceive themselves to be stereotyped, and that are therefore discouraged to invest in employability, social dialogue might be needed, as this can foster conversations on workers’ ambitions, perhaps opening up opportunities for labor market mobility (De Prins et al., 2015), which can intrinsically motivate them to continue their careers.

Second, policy-makers should realize that an inclusive approach to career development implies that workers’ different needs and values are respected and taken into consideration. Again, this requires social dialogue between workers and supervisors (cf. Euwema et al., 2015), but also between diverse (age) groups in the organization. When the different parties involved engage in career dialogues, workers and managers might be able to overcome (meta-)stereotypes they might hold about others and themselves and take opportunities they did not see for themselves (Bleijenbergh et al., 2016). Also supervisors might overcome stereotyped views regarding disadvantaged groups and learn to appreciate their various competences and talents (Meyers and Van Woerkom, 2014).

Third, to shape inclusive climates, all workers, regardless of their age, gender, ethnicity or intersections thereof (Mullings and Schulz, 2006), need to perceive that they are valued organizational members and have equal access to employability enhancing practices, which can satisfy their basic psychological needs (e.g., for belonging and uniqueness) (Nishii, 2013). This demands parties to develop more understanding and open communication in order to respond to different expectations regarding work, non-work and personal development, in line with individuals’ differences in talents and competences (Van der Heijden and De Vos, 2015).

Fourth, organizational stakeholders should monitor whether their HR policies and practices regarding recruitment, training and career development are not biased toward younger age groups, but are targeted at all age groups, as they can all add value to the organization (cf. Kunze et al., 2011).

## Conclusion

Older age group members in our study reported lower levels of employability, but particularly so when they perceived stronger negative stereotyping regarding their age group, which can be interpreted as meta-stereotyping. Because of the specific type of jobs that were incorporated in our empirical study, the psychological processes associated with age group membership and (meta-)stereotyping did not affect self-perceived occupational expertise, possibly because of the relatively low level of occupational knowledge and skills required to do the job. This may also explain the lack of significant effects on workers’ personal flexibility. However, in view of new labor market requirements and technological developments (e.g., ongoing automation), (lower-skilled) work becomes ever more uncertain, which also demands these workers to be prepared for making labor market transition. This stresses the growing importance of the employability dimensions anticipation and optimization and corporate sense for all workers. In order to reduce age-related (meta-)stereotyping, we argue that HR policies and practices, particularly social dialogue, can enhance inclusion by stimulating (future) ambitions among all workers, and disadvantaged workers in particular, and support them in creating career opportunities that can deliver psychological, social, and economic benefits.

## Ethics Statement

The data used in this paper was collected in the context of an internal, non-funded project conducted at the Business Administration Department of the Radboud University Nijmegen in Netherlands. According to the Regulations Ethics Committee, Faculty of Law and Nijmegen School of Management and national regulations, an ethics approval was not required. The instruction letter in the questionnaire, however, was in line with the criteria presented by the Regulations Ethics Committee, Faculty of Law and Nijmegen School of Management (https://www.ru.nl/law/research/ethics-committee/regulations). Besides informing all participants before their participation in the study that the data would be used for scholarly work, after data collection the team leader gave explicit permission for using the data for scholarly publication. Informed consent of the participants was implied through survey completion.

## Author Contributions

All authors listed have made a substantial intellectual contribution to the research. PP, BVdH, AV, and RK developed the theoretical conception. PP and DS developed the study design. DS performed the analyses and the drafted the figures and tables. PP and RK performed the data collection. PP, BVdH, DS, and AV interpreted the results and all contributed to drafting the paper.

## Conflict of Interest Statement

The authors declare that the research was conducted in the absence of any commercial or financial relationships that could be construed as a potential conflict of interest.
